# HIV-1gp120 Induces Neuronal Apoptosis through Enhancement of 4-Aminopyridine-Senstive Outward K^+^ Currents

**DOI:** 10.1371/journal.pone.0025994

**Published:** 2011-10-07

**Authors:** Lina Chen, Jianuo Liu, Changshui Xu, James Keblesh, Weijin Zang, Huangui Xiong

**Affiliations:** 1 Neurophysiology Laboratory, Department of Pharmacology and Experimental Neuroscience, University of Nebraska Medical Center, Omaha, Nebraska, United States of America; 2 Department of Pathology and Microbiology, University of Nebraska Medical Center, Omaha, Nebraska, United States of America; 3 Department of Pharmacology, College of Medicine, Xi'an Jiaotong University, Xi'an, People's Republic of China; Boston University School of Medicine, United States of America

## Abstract

Human immunodeficiency virus type 1 (HIV-1)-associated dementia (HAD) usually occurs late in the course of HIV-1 infection and the mechanisms underlying HAD pathogenesis are not well understood. Accumulating evidence indicates that neuronal voltage-gated potassium (Kv) channels play an important role in memory processes and acquired neuronal channelopathies in HAD. To examine whether Kv channels are involved in HIV-1-associated neuronal injury, we studied the effects of HIV-1 glycoprotein 120 (gp120) on outward K+ currents in rat cortical neuronal cultures using whole-cell patch techniques. Exposure of cortical neurons to gp120 produced a dose-dependent enhancement of A-type transient outward K+ currents (IA). The gp120-induced increase of IA was attenuated by T140, a specific antagonist for chemokine receptor CXCR4, suggesting gp120 enhancement of neuronal IA via CXCR4. Pretreatment of neuronal cultures with a protein kinase C (PKC) inhibitor, GF109203X, inhibited the gp120-induced increase of IA. Biological significance of gp120 enhancement of IA was demonstrated by experimental results showing that gp120-induced neuronal apoptosis, as detected by terminal deoxynucleotidyl transferase dUTP nick end labeling (TUNEL) assay and caspase-3 staining, was attenuated by either an IA blocker 4-aminopyridine or a specific CXCR4 antagonist T140. Taken together, these results suggest that gp120 may induce caspase-3 dependent neuronal apoptosis by enhancing IA via CXCR4-PKC signaling.

## Introduction

Human immunodeficiency virus type 1 (HIV-1)-infected individuals often suffer from neurological complications such as memory loss, mental slowing, and gait disturbance [1]. The severity of such impairments can vary, ranging from asymptomatic neurocognitive impairment, to mild neurocognitive disorder and to HIV-associated dementia (HAD), which are now collectively referred to as HIV-associated neurodegenerative disorders (HAND) [2]. The mechanisms of neuronal injury in HAND, including reduction of synaptic contacts [3], dendritic pruning [4], and selective neuronal loss [5] and apoptosis [6], remain incompletely understood. Current consensus holds that secreted soluble factors such as cytokines, chemokines, excitatory amino acids, nitric oxide, arachidonic acid and metabolites, and viral proteins, diffuse within the central nervous system (CNS) to directly or indirectly damage neurons [7], [8]. In particular, the role of HIV-1 envelope glycoprotein 120 (gp120) in HAND neuropathology has drawn considerable research attention.

In HIV-1-infected brain, gp120 may be shed off from virions or secreted as a soluble substance by HIV-1-infected mononuclear phagocytes. To model its effects in the CNS, gp120 was introduced into neuronal cultures and found to induce neuronal apoptosis [9], [10] even at very low concentrations [11]. This in vitro gp120-mediated apoptosis was then confirmed with ex vivo organotypic hippocampal slice preparations [12], transgenic over-expression of glial gp120 [13], [14], and direct stereotactic intracranial injection [15], [16]. A number of researchers have established that gp120-induced apoptosis can be prevented by blocking or down-regulating the CXCR4 receptors [17], [18], [19], indicating gp120 can induce apoptosis through the CXCR4. While research into the apoptotic pathways downstream of gp120 binding continue, new insights into the process of apoptosis are also being made, in particular with regard to the crucial role of voltage-gated potassium (Kv) channels.

Across various cell types, the process of apoptosis is characterized by cell volume decreases, caspase activation, and DNA fragmentation, with accumulating evidence demonstrating K+ homeostasis involvement in each stage [20], [21]. Since the original experiment by Shan Ping Yu, et al. [22] demonstrating K+ ionophore insertion was sufficient to initiate and sustain apoptosis in neurons, this sequence has been observed with various neuronal insults and accompanied by increased K+ channel current [23]. Furthermore, K+ channel blockade and high K+ medium have been found to prevent apoptosis in cultured cortical neurons [22], [24], [25], cerebellar granule neurons [26], [27], and rat hippocampal neurons [28]. Given the previous characterization of gp120-mediated apoptosis and the essential nature of K+ channel currents in the apoptotic process just described, here we test our hypothesis that gp120 induces neuronal apoptosis via enhancement of outward K+ currents. Our results demonstrated that gp120 increased 4-aminopyridine (4-AP)-sensitive, A-type transient outward K+ currents (IA) leading to neuronal apoptosis in rat cortical neuronal cultures.

## Materials and Methods

### Animal

Pregnant Sprague-Dawley rats were purchased from Jackson Laboratory (Bar Harbor, Maine) and maintained under ethical guidelines for care of laboratory animals at the University of Nebraska Medical Center. All animal-use procedures were reviewed and approved by the Institutional Animal Care and Use Committee (IACUC) of University of Nebraska Medical Center (IACUC # 00-062-07).

### Primary cortical neuronal culture

Purified cortical neurons were prepared from rat embryos described previously [29]. Briefly, female Sprague-Dawley rats with 18–19 days of gestation were anesthetized and embryonic pups were surgically removed. Cerebral cortices were harvested and digested using 0.25% trypsin and 200 U deoxyribonuclease I (Sigma-Aldrich, St. Louis, MO) in Hank's Buffered Salt Solution (HBSS) (Invitrogen, Carlsbad, CA) at 37°C for 15 min. After washing in HBSS, tissue mixtures were centrifuged, decanted and sequentially passed through a 100 µm- and 40 µm-mesh. The cells were seeded at a density of 5×10^5^/dish (or well) in poly-D-lysine-coated 35 mm culture dishes or 6 well plates and maintained in neurobasal medium (Invitrogen) supplemented with 2% B-27 serum-free supplement, 1% penicillin/streptomycin, 0.2% fetal bovine serum (FBS) and 0.25 mM L-glutamine (Invitrogen) for 7–10 days. The purity of neural cells was determined by staining with microtubule-associated protein-2 (MAP-2, a mature neuronal marker) antibody (Millipore, Temecula, CA) and more than 90% of MAP-2 positive cells were obtained.

### Electrophysiology

Whole-cell patch recordings were performed in rat cortical neuronal cultures in 35 mm tissue culture dishes on the stage of an inverted Nikon microscope (TE 300) using an Axopatch 200B amplifier (Molecular Devices, Sunnyvale, CA). Patch electrodes, made from borosilicate glass micropipettes (WPI Inc. Sarasota, FL) with a P-97 micropipette puller (Sutter Instruments, Novato, CA), had tip resistance of 5.0–8.0 MΩ. The electrodes were advanced towards cells by a Burleigh micromanipulator (PC-5000, EXFO, Canada). After establishment of the whole-cell patch configuration, the cells were allowed to stabilize for 3–5 min before tests. The recorded cells were held at −60 mV during voltage clamping. Whole-cell outward K^+^ currents were induced by applying 300 ms depolarizing steps from the holding potential of −60 mV to −40 mV in the first step, and then stepped to +60 mV in increments of 10 mV. Junction potentials were corrected, serial resistance was compensated and cell capacitance was partially (60–70%) compensated. Current signals were filtered at 1 kHz and digitized at 5 kHz using Digidata 1440A digitizer (Molecular Devices). The current and voltage traces were displayed and recorded in a computer using pCLAMP 10.2 data acquisition/analysis system (Molecular Devices).

The pipette solution for voltage-clamp experiments contained (in mM): 108.0 K_2_HPO_4_, 9.0 HEPES, 9.0 EGTA, 2.5 MgCl_2_, 14.0 creatine phosphate (Tris salt), 1.0 Mg-ATP, and 0.3 Tris-GTP, buffered to pH 7.4 with KOH. The extracellular solution contained (in mM): 150.0 NaCl, 4.0 KCl, 2.0 MgCl_2_, 2.0 CoCl_2_, 10.0 HEPES, 20 tetraethylammonium (TEA, Sigma-Aldrich) and 10 glucose, buffered to pH 7.4 with NaOH. In order to block sodium channels, 0.3 µM tetrodotoxin (TTX; Tocris, Ellisville, MO) was added. To block calcium-activated K^+^ currents, extracellular Ca^2+^ was replaced with equimolar Co^2+^ (2.0 mM) [30]. Stock solutions of TEA (1.0 M), 4-AP (1.0 M, Sigma-Aldrich), and TTX (0.2 mM) were prepared in deionized water and either stored at 4°C (TEA and 4-AP) or in aliquots at −20°C (TTX).

All experiments were done at room temperature (22–23°C). The neuronal cells were identified by their triangular-shaped morphology and their firing of action potentials in response to a depolarizing current injection. Chemical reagents were applied through incubation (2 h) in 95% CO_2_ and 5% O_2_ at 37°C. Data were analyzed by Clampfit 10.2 (Molecular Devices). For each set of experiments, the instantaneous outward currents generated by voltage steps from −60 mV to +60 mV were measured and analyzed.

### TUNEL assays

Terminal deoxynucleotidyl transferase dUTP nick end labeling (TUNEL) staining was performed to evaluate apoptotic neurons by using *in situ* cell death detection kit, *AP* (Roche Applied Science, Indianapolis, IN) according to the manufacturer's instructions. Briefly, rat cortical neurons grown on poly-D-lysine coated coverslips at a density of 1×10^5^/well in 24 well plates were pre-treated with CXCR4 blocker, T140 (50 nM, kindly provided by Dr. Nobutaka Fujii, Graduate School of Pharmaceutical Sciences, Kyoto University, Kyoto 606-8501, Japan) or K_v_ channel blocker 4-AP (5 mM) 30 min before addition of 500 pM gp120 (ImmunoDiagnostics, Inc., Woburn, MA). After treatments of 24 h, the neurons were fixed with 4% paraformaldehyde and permeabilized 0.1% Triton X-100 (Sigma) in 0.1% sodium citrate solution for 2 min on ice. After wash, the cells were then incubated with TUNEL reaction mixture containing terminal deoxynucleotidyl transferase and fluorescein-labeled nucleotides at 37°C for 60 min. After final wash, coverslips were mounted in ProLong Gold antifade reagent (Molecular Probes, Eugene, OR) with 4′,6′-diamidino-2-phenylindol (DAPI) and visualized by fluorescent microscope using a ×20 objective. The TUNEL-positive cells were analyzed by NIH ImageJ software and the percentage of TUNEL-positive cells (green) was normalized to total DAPI-positive cells from 12 microscopic fields.

### Immunocytochemistry

Neurons growing on poly-D-lysine-coated coverslips were treated with gp120 in the presence or absence of T140, 4-AP, or as indicated. After 24 h treatments, neurons were fixed in 4% paraformaldehyde and blocked in phosphate buffered saline (PBS) containing10% normal goat serum and 0.1% Triton X-100 for 30 min at room temperature. Neurons were incubated with primary antibody anti-caspase 3 (1∶200 Cell Signaling Technology, Beverly, CA) or mouse monoclonal anti-NeuN (1∶100; Millipore) diluted in blocking solution for 2 h, followed by application of AlexaFluor 488 (1∶500) and AlexaFluor 594 (1∶500) secondary antibody (Invitrogen) in PBS+10% HIGS for 1 h. The fluorescent images were captured using Olympus DP70 camera and DP Controller Ver. 2.1.1 software. The values of fluorescent intensity were acquired using ImageJ software (National Institutes of Health) and then normalized with cell numbers.

### Statistics

All data were expressed as mean ± SEM and graphed using Origin 8.0 software (OriginLab, Northampton, MA) unless otherwise indicated. Statistical analyses were performed by one-way ANOVA analysis or by Student *t* tests. A minimum p value of 0.05 was estimated as the significance level for all tests.

## Results

### Expression of outward K^+^ currents in cultured rat cortical neurons

**Figure 1:**
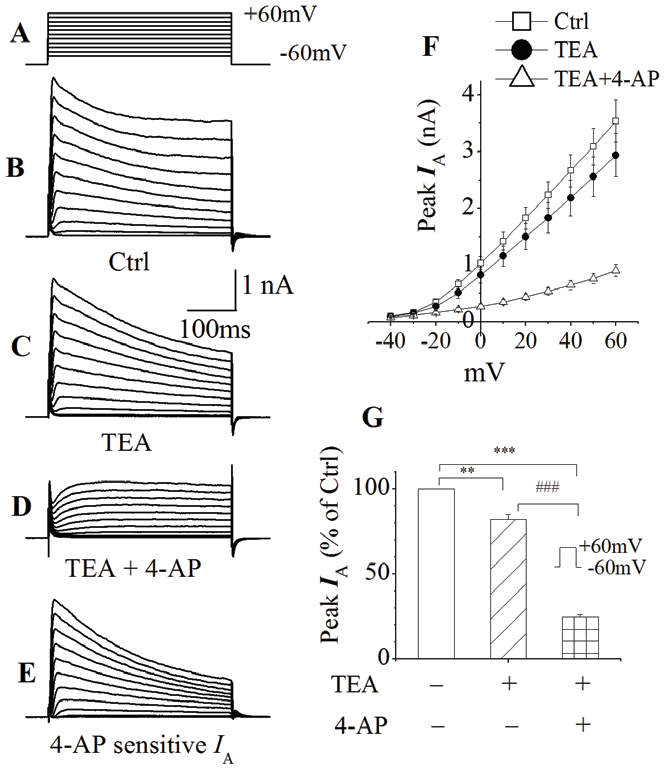
Expression of outward K^+^ currents in cultured rat cortical neurons. Whole-cell outward K^+^ currents (B) was induced by voltage steps (300 ms in duration) from the holding potential of −60 mV to −40 mV in the first step, and then stepped to +60 mV in increments of 10 mV as shown at the top (A) (This voltage protocol was used throughout this study). Addition of TEA (20 mM) to the bath reduced the instantaneous current (C) and the “TEA-resistant” current was further reduced by addition of 5 mM 4-AP (D). 4-AP-sensitive outward K^+^ currents (*I*_A_) were obtained by subtraction of the outward K^+^ currents shown in D from the outward K^+^ currents illustrated in C (E). F depicts the I–V curves as indicated. G illustrates the normalized peak outward K^+^ currents taken at +60 mV showing that TEA produced approximately 17% of reduction of peak outward K^+^ currents while 4-AP yielded about 58% of reduction. Values are presented as the mean ± SEM, n = 8. **p<0.01, ***p<0.001, ### p<0.001.

Outward K^+^ currents generated by voltage steps (Fig. 1A) were recorded in rat cortical neurons after 7–10 days in culture. The average peak current amplitude, generated by a voltage step from −60 mv to +60 mV, was 3541.9±369.5 pA (Fig. 1B, n = 8). Addition of TEA (20 mM) to the bath solution reduced the peak current to 2940.6±383.5 pA (Fig. 1C, n = 8), producing a reduction of ∼17% on peak current. The peak current was further reduced to 905.3±110.8 pA (Fig. 1D, n = 8) by addition of 4-AP (5 mM) to the bath solution, resulting in a reduction of ∼69% on the remaining peak current. The 4-AP-sensitive outward K^+^ currents were isolated by subtraction of the currents recorded in the presence of both TEA and 4-AP from those recorded in the presence TEA alone (Fig. 1E). Thus, the reduction on peak current produced by 4-AP was ∼58% when measured at the voltage step of +60 mV.

### Enhancement of neuronal I_A_ by gp120

**Figure 2:**
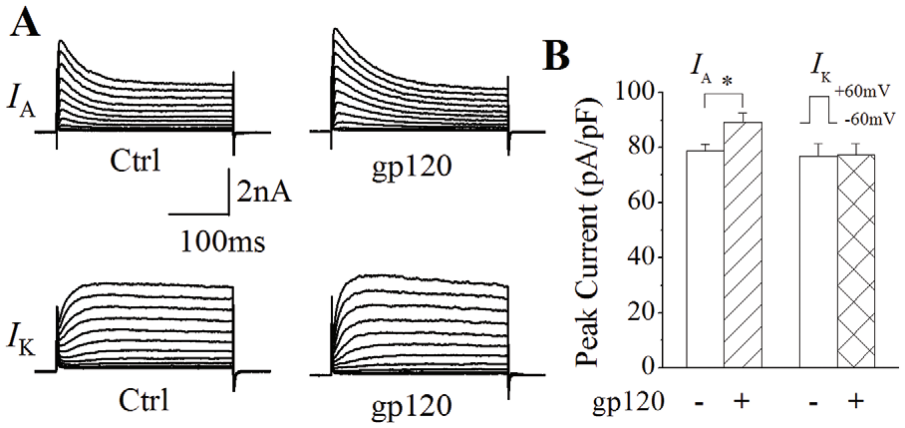
Enhancement of *I*_A_ by gp120. A shows representative current traces recorded in control (Ctrl, left) and gp120-treated (gp120, right) rat cortical neurons in the presence of TEA (20 mM, upper) and 4-AP (5 mM, lower). The voltage protocol utilized to generate outward K^+^ currents were the same as shown in Fig. 1A. Note that gp120 enhanced *I*_A_ (upper) with no apparent effect on delayed rectifier like *I*_K_ (lower). B is a summarized bar graph illustrating gp120 enhancement of the *I*_A_, but not the *I*_K_. Each value represents the mean ± SEM. * *p*<0.05; gp120 (n = 115) vs Ctrl (n = 128) for *I*_A_. *p*>0.05; gp120 (n = 35) vs Ctrl (n = 30) for *I*_K_.

**Figure 3:**
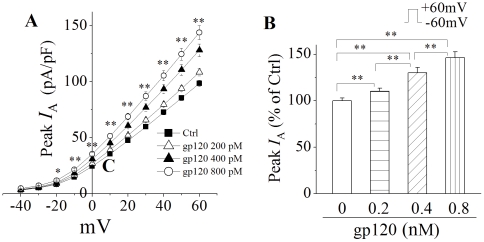
gp120 increased neuronal *I*_A_ in a dose-dependent manner. Panel A illustrates the I–V plots of *I*_A_ current densities in the absent and present of gp120 at different concentrations indicated. * *p*<0.05, ** *p*<0.01, gp120 200 pM (n = 115), 400 pM (n = 63), 800 pM (n = 57) vs Ctrl (n = 128), respectively. Panel B is a bar graph showing the average percentage of gp120-induced increase of neuronal *I*_A_ when the instantaneous peak currents generated in response to +60 mV voltage step were measured. ** *p*<0.01 as indicated.

It is well-known that gp120 induces neuronal apoptosis [8] and that enhancement of outward K^+^ currents is believed to be an essential pathway in programmed cell death [22], [24], [25]. To examine whether gp120 induces neuronal apoptosis via increasing *I_A_*, we first tested the effects of gp120 on *I_A_* in primary rat cortical neuronal cultures. The 4-AP-sensitive *I_A_* and the 4-AP-insensitive delayed rectifier like outward K^+^ currents (*I*_K_) were recorded by addition of TEA (20 mM) and 4-AP (5 mM) to the extracellular solution, respectively. Incubation of rat cortical neurons with gp120 (200 pM) for 2 h produced an enhancement of *I_A_*, with an average instantaneous current density of 89.2±3.4 pA/pF (n = 115). In comparison with the current density of 78.7±2.6 pA/pF (n = 128) recorded in control neurons (without gp120 incubation), the difference was statistically significant (Fig. 2, *p*<0.05). In contrast, the *I_K_* density recorded in neurons with and without incubation with gp120 were 77.4±4.0 pA/pF (n = 35) and 76.8±4.6 pA/pF (n = 30), respectively. The difference was not statistically significant (Fig. 2, *p*>0.05). These results indicate that gp120 differentially enhances *I*_A_ in cultured rat cortical neurons. The enhancement of *I*_A_ induced by gp120 was dose-dependent, with average current densities of 89.2±3.4 pA/pF (n = 115), 102.6±4.5 pA/pF (n = 63), 115.3±5.0 pA/pF (n = 57) when neuronal cells were incubated with gp120 at concentrations of 200, 400 and 800 pM, respectively. The differences are statistically significant (*p*<0.01), demonstrating an enhancement of *I*_A_ by gp120 (Fig. 3).

### gp120 increases I_A_ via CXCR4

**Figure 4:**
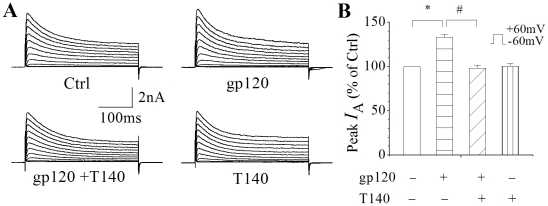
gp120 increases neuronal *I*_A_ *via* CXCR4. A, Representative *I*_A_ current traces recorded in the presence of 20 mM TEA from a control neuron (Ctrl) and neurons incubated with 200 pM gp120 (gp120), gp120+T140 (gp120+T140) and 50 nM T140 (T140), respectively. B, Bar graph showing the average instantaneous peak current amplitude (% of control) generated by a voltage step from −60 mV to 60 mV. Note gp120 produced a significant increase of peak *I*_A_ and this increase was blocked by T140, a CXCR4 receptor antagonist, indicating gp120 increase of neuronal *I*_A_ via CXCR4. * *p*<0.05, gp210 (n = 115) vs Ctrl (n = 128); # *p*<0.05, gp120 (n = 115) vs gp120+T140 (n = 70).

C-X-C chemokine receptor type 4 (CXCR-4), a co-receptor for HIV-1 infection, is expressed in the brain in a variety of cell types including neurons [8], [31], [32]. Studies have shown that gp120 induces neuronal injury via CXCR4 [17], [33]. To assess whether gp120-induced enhancement of *I*_A_ is mediated through activation of neuronal CXCR4, we studied the effects of T140, a specific CXCR4 receptor antagonist [34], [35], on its blockade of gp120-induced enhancement of *I*_A_. As shown in Fig. 4, incubation of cortical neurons with gp120 (200 pM) increased the *I*_A_ density by 33.4%, from 78.7±2.6 pA/pF (n = 128) to 89.2±3.4 pA/pF (n = 115). Addition of T140 (50 nM) to the incubation medium blocked gp120 enhancement of the *I*_A_, with an average current density of 77.4±2.5 pA/pF (n = 70). In comparison with the gp120-induced enhancement, the difference is statistically significant (*p*<0.05), suggesting gp120 increase of neuronal *I*_A_ via CXCR4. Application of T140 alone had no apparent effect on neuronal *I*_A_ (Fig. 4).

### Involvement of PKC signaling in gp120-mediated increase of I_A_

**Figure 5:**
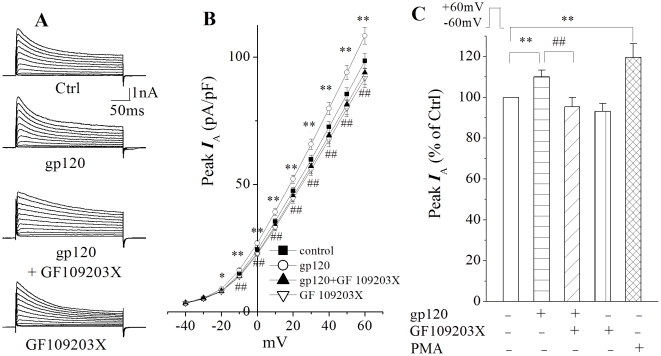
Involvement of PKC in gp120-mediated enhancement of neuronal *I*_A_. A, *I*_A_ currents recorded in the presence of 20 mM TEA from a control neuron and neurons incubated with gp120 (200 pM), gp120+GF109203X (5 µM, a PKC inhibitor), and GF109203X(5 µM), respectively. B I–V curves illustrating *I*_A_ current densities generated by voltage steps in control neurons (n = 128) and neurons treated with gp120 (n = 115), gp120+GF109203X (n = 57) and GF109203X alone (n = 55). * *p*<0.05, ** *p*<0.01, gp120 vs Ctrl; ## *p*<0.01, gp120+GF109203X vs gp120. C is a bar graph plotting the average peak *I*_A_ currents (% of ctrl) measured at +60 mV. Note that gp120 enhanced neuronal *I*_A_ and this enhancement was blocked by a specific PKC inhibitor GF109203X. Incubation of cortical neurons with PMA, a PKC activator (100 nM, n = 49), for 30 min also produced a significant enhancement of neuronal *I*_A_, suggesting that PKC pathway is involved in gp120-mediated enhancement of neuronal *I*_A_. ** *p*<0.01 and ## *p*<0.01 for comparisons as indicated.

As an extracellular toxic molecule, gp120 represents one of the many exogenous signals that must be integrated by neurons. The neurons accomplish this through evolutionarily conserved signaling cascades, often comprised of reversibly modifiable kinases such as the protein kinase C (PKC) family of isozymes [36]. PKC can be activated by G-protein coupled receptors such as CXCR4 through production of its direct activator diacylglycerol (DAG) and can have either pro- or anti-apoptotic effects depending on the particular stimuli, cell type, and isozyme activated [37], [38], [39]. Recently, PKC activation was shown to be involved in increasing *I*_A_ and neuronal apoptosis in rat cerebellar granule neurons [27]. In order to determine whether gp120 increases *I*_A_ through activation of PKC, we used phorbol myristate acetate (PMA) to mimic DAG activation of PKC and GF109203X as a PKC inhibitor. Figure 5 shows brief incubation of rat cortical neurons with PMA produced an increase of *I*_A_ similar to those produced by gp120, with an average current density of 94.4±5.2 pA/pF (n = 49). The difference is statistically significant (p<0.01) when compared to the average current density of 78.7±2.6 pA/pF (n = 128) recorded in control neurons, indicating an increase neuronal *I*_A_ via PKC. The involvement of PKC signaling in gp120-mediated enhancement of neuronal *I*_A_ was demonstrated by experimental results showing that co-incubation of cortical neurons with gp120 and PKC inhibitor GF109203X significantly (p<0.01) attenuated gp120-associated increase of *I*_A_. The average current densities recorded in the absence and presence of GF109203X were 89.2±3.4 pA/pF (n = 115) and 75.2±3.7 pA/pF (n = 57), respectively.

### Inhibition of gp120-induced apoptosis by 4-AP and T140

**Figure 6:**
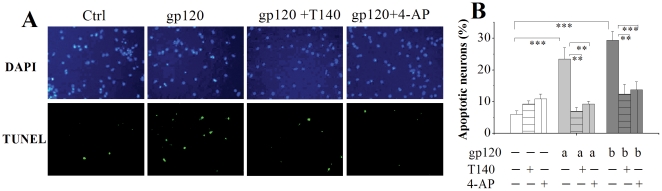
Attenuation of gp120-induced neuronal apoptosis by T140 or 4-AP. A shows immunocytochemical analysis of apoptosis (TUNEL staining) in cortical neuronal cultures induced by gp120 (200 pM) in the absence and presence of T140 (50 nM) or 4-AP (5 mM). Intact cell nuclei were visualized with DAPI staining (blue) of nucleic acids and apoptotic cells were labeled with TUNEL staining (green) of fragmented DNA (magnification ×40). B is a bar graph illustrating the percentage of TUNEL positive cells in response to gp120, T140 and 4-AP and showing that cultures incubated with gp120 for 24 h exhibited a significant increase of apoptotic neurons and that application of T140 or 4-AP significantly attenuated the gp120-induced increase of neuronal apoptosis. 12 randomly selected visual fields were counted in each group ** *p*<0.01, *** *p*<0.01. a; 200 pM; b; 500 pM.

Activation of K_v_ channel has been considered an essential pathway in programmed cell death [21]. To investigate whether the gp120-mediated enhancement of *I*_A_ contributes to gp120-induced neuronal injury, we examined the protective effects of 4-AP on gp120-induced neuronal apoptosis in rat cortical neuronal cultures. Cell nuclei were labeled with DAPI staining (blue) and apoptotic cells were determined by TUNEL staining (green) of fragmented DNA (Fig. 6A). Visualized by TUNEL staining, incubation of neuronal cultures with gp120 for 24 h resulted in neuronal apoptosis in a dose-dependent manner as shown in Fig. 6B. At the concentrations of 200 pM and 500 pM, the percentages of apoptotic neurons induced by gp120 were 23.4±3.6% and 29.3±2.8% (n = 4, each in triplicate, the same in the followings of this section), respectively, compared to 6.1±1.0% (n = 4) of apoptotic cells observed in control, the difference was statistically significant (Fig. 6), demonstrating gp120 induces neuronal apoptosis in primary rat neuronal cultures. The gp120-induced neuronal apoptosis was partially blocked either by 4-AP or the CXCR4 antagonist T140 (Fig. 6). In one subset of cortical neuronal cultures co-incubated with 4-AP (5.0 mM) and gp120 (200 pM or 500 pM), the average percentages of apoptotic neurons were 9.3±0.9% and 13.8±2.5%, respectively. In comparison with the average percentage of apoptotic neurons observed in neuronal cultures incubated with gp120 alone, the differences were statistically significant (Fig. 6, p<0.01, n = 4), indicating that involvement of *I*_A_ in gp120-induced neuronal apoptosis. In another subset of cortical neuronal cultures co-incubated with T140 (50 nM) and gp120 (200 pM or 500 pM), the average percentage of apoptotic neurons was significantly (p<0.01, n = 4) decreased to 7.0±1.2% and 12.4±3.1% (Fig. 6), respectively, further supporting our aforementioned findings that gp120 enhances neuronal *I*_A_ via CXCR4.

### Blockade of I_A_ inhibits gp120-induced caspase-3 activation

**Figure 7:**
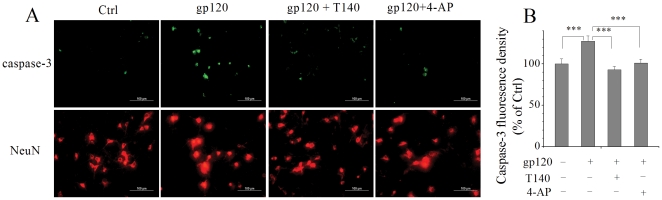
Activation of caspase-3 is involved in gp120 enhancement of neuronal *I*_A_ and resultant neuronal apoptosis. A, Photomicrograph of neuronal cultures treated with gp120 in the absence or presence of T140 or 4-AP. Caspase-3 was labeled with anti-caspase-3 antibody (green) and nuclei were labeled with NeuN (red). Note that gp120 increased the caspase-3 positive cells and this increase was attenuated by either T140 or 4-AP. Twelve different areas were measured in each group, and the experiments were done in triplicates (magnification ×40). B, A bar graph illustrates the average fluorescence density of caspase-3 detected in different experimental conditions. The fluorescence intensity in the control (Ctrl) group was normalized as 100%. *** *p*<0.001.

It has been shown that gp120 induces neuronal injury via activation of caspase-3 [13], [19], [40]. Our TUNEL results revealed that gp120 enhancement of *I*_A_ underlies gp120-induced neuronal apoptosis as blocking *I*_A_ decreased gp120-associated neuronal apoptosis. We further investigated if caspase-3 is the downstream pathway of gp120 enhancement of *I*_A_ in rat cortical neurons. Caspase-3 was detected with anti-caspase 3 staining (green) and neurons were marked with anti-NeuN staining (red). Neuronal cultures incubated with gp120 for 24 h exhibited a robust caspase-3 activation (Fig. 7A) with an average fluorescence density of 127.4±6.6% of non-gp120-treated control (p<0.001). This gp120-associated increase of caspase-3 fluorescence was significantly reduced by addition of 4-AP (5.0 mM) to the incubation media, with an average of 101.2±4.7% (Fig. 7B). These results suggest that gp120-mediated enhancement of *I*_A_ may cause caspase-3 activation and consequent neuronal apoptosis.

## Discussion

The mechanisms underlying HAND pathogenesis are at present incompletely understood. Over the years, the neurotoxicity of the viral protein gp120 has been established and several mechanisms proposed including N-methyl-D-aspartic acid (NMDA) receptor-mediated excitotoxicity and CXCR4 signaling [8]. To our knowledge, no work has been undertaken to connect gp120 toxicity to the excessive efflux of K^+^ necessary for apoptosis to occur. The critical finding of this study is the ability of gp120 to increase outward K^+^ currents in cultured rat cortical neurons. More specifically, the peak amplitude of *I*_A_ was increased in a dose-dependent manner and could be blocked, along with gp120-induced apoptosis, by simultaneous treatment of gp120-incubated cortical neurons with the K_v_ channel blocker 4-AP.

Several pieces of additional data collected point towards a possible mechanism. First, the CXCR4 inhibitor T140 was able to block both gp120-mediated increases in *I*_A_ and apoptosis. Given the accumulation of research demonstrating a role for CXCR4 signaling in gp120 apoptosis [17], [41], the effects on apoptosis are perhaps unsurprising. To our knowledge, however, this is the first work demonstrating outward K^+^ currents can be increased through CXCR4 signaling. Combined with ample research having established a critical role for K_v_ channel in initiating and sustaining apoptosis [22], [25], these results may perhaps indicate a toxic mechanism of gp120 that has thus far been unappreciated. It should be noted that while many of the experiments regarding apoptotic K^+^ current have focused on sustained delayed rectifier currents, there have been other reports of 4-AP-sensitive outward K^+^ currents contributing to apoptosis [26], [42], [43]. In any case, it is the chronic efflux and intracellular depletion of K^+^ that is necessary for apoptosis, which in pathological conditions could occur *via I*_A_. Our finding that gp120 increases *I*_A_ and that 4-AP blocks gp120-induced apoptosis stands as evidence of this possibility. Since our evidence indicates this occurs *via* the G-protein coupled CXCR4 receptors, we next attempted to identify an intracellular signaling cascade, such as PKC, that could be responsible for this pathological increase in *I*_A_.

In general, PKC activation occurs through G-protein coupled receptors, which when signaled activate phospholipase C (PLC) to hydrolyze phosphatidylinositol-4,5-bisphosphate (PIP_2_) to DAG [38], [44]. PKC isozymes can be categorized according to the combination of phosphatylserine (PS), DAG, and Ca^2+^ required for activation as conventional (PS, DAG, and Ca^2+^), novel (PS and DAG), or atypical (PS) [44]. In this experiment, we used the phorbol ester PMA to mimic DAG activation of conventional and novel isozymes. We found this activation of PKC to mimic gp120 increases in *I*_A_. However, precision targeting of PKC isoforms has revealed that, in general, conventional isoforms (such as PKCα) have anti-apoptotic roles while novel isoforms (such as PKCδ) have pro-apoptotic roles [37], [39]. The relative expression of different PKC isozymes also varies with tissue, subcellular localization, and even between neuronal compartments [38], complicating the situation. Therefore, while PKC activation has been shown elsewhere to decrease K_v_ channel current [45], [46], [47] and in some cases has neuroprotective effects [48], [49], PKC activators have been shown to induce apoptosis in many cell types [50], [51], [52], [53] including neurons [27], [54] and chemical inhibition or mutation of apoptotic PKC isozymes in many cases attenuates this apoptosis [50], [54], [55], [56]. Under our experimental conditions, the apoptotic effects of brief exposure to PMA were confirmed by assay of reactive oxygen species (data not shown). Furthermore, PKC inhibition using GF109203X was protective against gp120-mediated apoptosis. While not definitive, these experiments lay the groundwork for understanding the intracellular signals involved in apoptotic gp120 increases in *I*_A_ and indicate a possible role of apoptotic PKC pathways.

With our other mechanistic studies, the emerging picture indicates a possible scenario whereby gp120 affects apoptosis through G-protein-coupled CXCR4 receptor signaling and activates apoptotic PKC–caspase-3 pathways. Overall, the finding that gp120 mediates enhancement of *I*_A_ could have relevance at several levels within the context of HAND. The first and most obvious biological significance is the contribution of enhancement of *I*_A_ to gp120-induced apoptosis. Current consensus holds that HIV-infected and activated macrophages migrate across a weakened blood-brain barrier and secrete soluble viral proteins in addition to cellular factors that lead to direct or indirect neuronal damage [8]. Of these viral proteins, substantial evidence has implicated gp120 in the neurotoxic brain pathology underlying HAND [10], [13], [15]. Gp120-induced neuronal damage has been linked to NMDA receptor excitotoxicity and CXCR4 receptor [8]; however, until now, the involvement of K^+^ homeostasis in the apoptotic process [20], [21], [23] in association with HIV-1-associated neurodegeneration has largely been unappreciated. Bringing together these two avenues of research may help complete the mechanism of gp120-induced neurotoxicity and HAND neuropathology.

Secondly, cognitive decline in HAND may result as much from neuronal dysfunction as from neuronal loss, an idea supported by experimental results showing alterations in cell layer volume [57] and dendritic morphology [4] correlate with HAD [58]. Indeed, extensive cell death is not always present when symptoms manifest [59] and antiretroviral therapy (ART) treatment has been known to lead to cognitive improvement [60], [61], [62], [63], suggesting the underlying pathology of HAD may be in part reversible. This is consistent with a channelopathy hypothesis originally described by Dr. Ben Gelman [64], which led our laboratory to investigate K_v_ channel involvement in HAND [65]. K_v_ channels are well known to regulate membrane potential and thereby the repolarization, discharge frequency, and waveforms of action potentials (AP). Due to a negative equilibrium potential relative to the AP threshold, outward K^+^ currents are essentially inhibitory. Not surprisingly, decreased outward K^+^ currents have been found to correspond with improved memory and long-term potentiation (LTP), while increased outward K^+^ currents correspond to learning and memory deficiencies [65]. Continuing research involving outward K^+^ currents has given broad support to this general concept. Whether dendritic, somatic, axonal, or terminal, K_v_ 1.1 (with β subunit), K_v_ 1.4, or K_v_ 4, appropriate A-type current has been shown to be crucial for LTP induction, learning and memory, axonal signal propagation, and terminal transmitter release [65]. Therefore, increased outward K^+^ currents could be expected to have a deleterious effect on neuronal function with subsequent induction of apoptosis. In previous experiments, we found injection of HIV-infected macrophages into severe combined immune deficient (SCID) mice basal ganglia inhibits LTP and impairs radial arm water maze performance, measures of learning and memory that were restored with systematic administration of the K_v_ channel blocker 4-AP [66]. The present research more specifically implicates gp120-induced increases in *I*_A_ as the underlying mechanism of neuronal dysfunction and eventual cell death in HAND.

While gp120 is supposed to increase *I*_A_ by direct action on neurons, it should be noted that 5–10% of the cells present in culture are glia. Gp120 can also interact with glia and promote the release of immunological factors [67], [68] that could also potentially alter outward K^+^ currents [65]. Previous experiments in our laboratory have demonstrated that conditioned media recovered from immune-activated macrophages increase neuronal outward K^+^ currents and induce neuronal apoptosis [69] suggesting the gp120 increases of neuronal outward K^+^ currents may be in some part attributable to indirect activation of bystanders such as glial cells and resultant production of soluble substances. This issue is significant in the sense of understanding the precise mechanism of gp120-induced increases in *I*_A_ and apoptosis. However, the key finding here remains that gp120 does indeed induce enhancement of neuronal outward K^+^ currents and apoptosis, which can be attenuated by K_v_ channel blocker 4-AP. While many questions still exist, knowledge of K_v_ channel dysfunction induced by gp120 and other soluble factors can serve as a starting point for developing adjunctive therapies to target the disrupted neuronal function in HAND.

**Figure 8:**
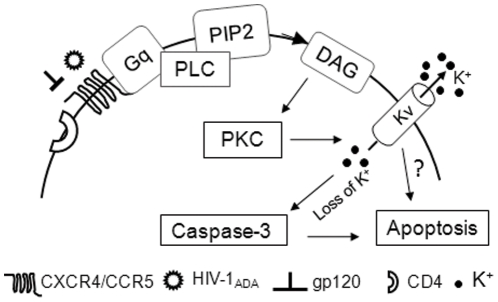
A schematic diagram illustrating the potential pathways for gp120 enhancement of neuronal *I*_A_ and resultant neuronal apoptosis.

In summary, the experimental data provide *in vitro* evidence that the HIV-1gp120 increases *I*_A_ via CXCR4-PKC pathway leading to neuronal apoptosis. The enhancement of *I*_A_ resulted in neuronal apoptosis by activation of caspase-3 (Fig. 8) and the gp120-induced neuronal apoptosis was significantly attenuated by 4-AP, a K_v_ channel antagonist. These results suggest that K_v_ channels are involved in HIV-1-associated neuropathogenesis.
